# Potential Association between Zika Infection and Microcephaly during 2007 Fever Outbreak, Gabon 

**DOI:** 10.3201/eid2702.202987

**Published:** 2021-02

**Authors:** Claudine A. Kombila Koumavor, Eric Elguero, Eric M. Leroy

**Affiliations:** University of Health Sciences, Libreville, Gabon (C.A. Kombila Koumavor);; Institute for Sustainable Development, IRD-CNRS-University of Montpellier Unit, Montpellier, France (E. Elguero, E. Leroy)

**Keywords:** Aedes albopictus, Africa, febrile illnesses, Gabon, high-risk pregnancy, microcephaly, outbreaks, vector-borne infections, viruses, Zika, Zika virus

## Abstract

Although Zika virus (ZIKV) circulates in sub-Saharan Africa, no case of ZIKV-associated microcephaly has thus far been reported. Here, we report evidence of a possible association between a 2007 outbreak of febrile illness and an increase in microcephaly and possibly ZIKV infection in Gabon.

Since its 1947 discovery in Uganda, Zika virus (ZIKV) was restricted to sporadic human infections in Africa and Asia until 2007, when a large outbreak occurred in Micronesia, followed by another in French Polynesia 6 years later. This second outbreak spread to Brazil and throughout Central and South America, resulting in hundreds of thousands of cases (*1*). ZIKV infection leads to an asymptomatic or mildly symptomatic nonspecific disease in 80% of cases, but the outbreak in the Americas and French Polynesia coincided with a steep increase in the birth of babies with congenital microcephaly (*2*–*4*). However, no case of ZIKV-associated microcephaly has been recorded in sub-Saharan regions of Africa, where ZIKV also circulates. 

During April–August 2007, Gabon’s capital, Libreville, experienced simultaneous outbreaks of chikungunya and dengue (*5*). A retrospective study of 4,312 serum samples collected during this time found 5 ZIKV-positive cases (*6*). In addition, 2/137 (1.46%) pooled samples from *Aedes albopictus* mosquitoes tested positive for ZIKV, a proportion similar to that observed for dengue virus. Given that 80% of ZIKV infections are asymptomatic or subclinical, these findings suggest that an undetected ZIKV outbreak may have occurred in Gabon in 2007. 

To determine if the incidence of microcephaly increased during this suspected ZIKV outbreak, we examined birth registers at the 2 main hospitals of Libreville: the Libreville Hospital Centre and the Regional Hospital of Melen in Estuaire Province. We recorded all births and cases of microcephaly occurring during January 2006–December 2008 (Figure). Most births in Libreville and its suburbs occur in these 2 hospitals; in addition, the hospitals receive newborns with malformations observed at birth who have been transferred from smaller healthcare facilities that lack neonatal departments. We collected most of the 4,312 samples from patients who visited these hospitals, so the 5 ZIKV case-patients likely lived in the 2 hospitals’ coverage area. 

**Figure:**
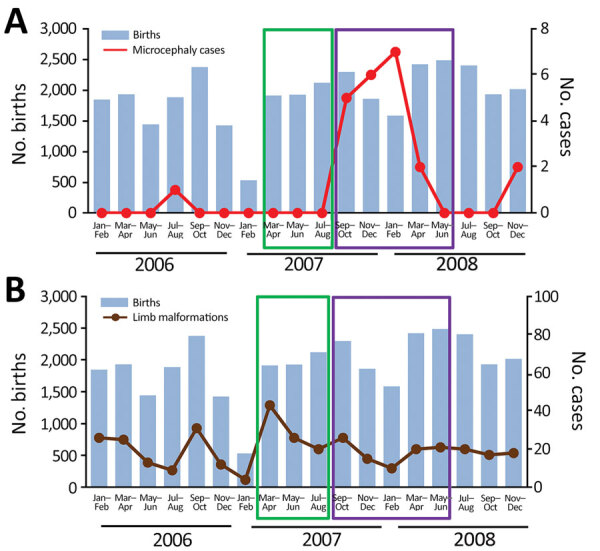
Cases of microcephaly (A) and limb malformations (B) in Libreville, Gabon, during January 2006–December 2008. Histograms correspond to the total number of births over the time of the study period. Scales for the y-axes differ to underscore patterns but do not permit direct comparisons. Green boxes encompass the time period when the febrile illness outbreak happened; purple boxes encompass the time period of births of infants whose mothers could have been exposed to Zika in their first trimester of pregnancy during the outbreak. Numbers of births were collected from birth registers by 2-month periods from January–February 2006 through November–December 2008. (Births are recorded in the registers in such a way that we were unable to obtain data for single months.) The duration of the febrile illness outbreak was estimated by the Health Ministry of Gabon during April 2007–August 2007, based on information communicated by hospitals. The febrile illness outbreak coincided with an increase in the number of patients seeking treatment for painful febrile illnesses at healthcare centers beginning in April 2007. The end of the outbreak in August 2017 coincided with the disappearance for >15 days of grouped clinical cases and with negative test results from samples.

In 2017, we searched birth registers for cases of microcephaly, identified when the head circumference was 2 SDs below the average, according to World Health Organization standards, depending on the age and sex of the neonate. For male-born infants, microcephaly corresponded to a cranial circumference of <31.9 cm, and for female-born infants, a cranial circumference of <31.5 cm, measured <48 hours after birth. We recorded only data from physical examination of newborns. 

We collected details of 34,409 births and grouped them by 2-month periods from January–February 2006 through November–December 2008. Children were considered exposed if they were born during May 2007–June 2008 to mothers pregnant during April 2007–August 2007, as described elsewhere (*7*). We calculated statistical significance using the ratio of the odds of an infant with microcephaly being born within or outside of the exposure period. Only 1 case of microcephaly was recorded during January 2006–April 2007 (Figure, panel A), suggesting a baseline rate of ≈1 case/year. 

Among 10,286 children born in the 14 months during May 2007–June 2008, a total of 20 microcephaly cases were recorded, compared with only 2 cases among 24,123 children born in the 20 months of the study period outside of the outbreak (OR 15.6, 95% CI 4.65–52.70; p = 8.8 × 10^–6^; Figure, panel A). In contrast, we found no increase in newborns with other types of malformation, such as limb malformations, during that period (Figure, panel B). Of note, 18 of the 20 outbreak-associated children with microcephaly were born during September 2007–February 2008, corresponding to mothers in the first trimester of pregnancy during the outbreak, when the risk of microcephaly in fetuses or neonates is highest. To eliminate potential artifacts in the data arising from unspecified environmental incidents, we used the same method of analysis to examine other congenital birth malformations such as facial, upper limb, and lower limb malformations. No significant associations were found. 

Limitations of our study included that the tabulations (conducted in 2017) of ZIKV infections (from the 2007 disease outbreak) and microcephalic births were retrospective, meaning that no investigation of ZIKV infection was performed during the fever outbreak. Thus, there was no ZIKV diagnosis at the time of delivery for any of the mothers of infants born with microcephaly. Although fetal malformations are sometimes detected in obstetric ultrasounds, in Gabon they are usually discovered at birth. In addition, this study did not directly investigate the etiology of birth malformations for other possible explanations.

Despite these limitations, our findings support that the 2007 febrile illness outbreak in Libreville was associated with an increase in infants with microcephaly. Although microcephaly may be due to many other causes that were not investigated in our study, the detection of 5 ZIKV cases in samples collected during the febrile illness outbreak suggests a temporal association between ZIKV and microcephaly in this country. Given the risk of microcephaly in infants is ≈1% for mothers infected during the first trimester of pregnancy (*8*), the high number of microcephaly cases reported here indicates that ZIKV infections were likely prevalent during the outbreak. These observations highlight the need to provide specific priority care for pregnant women during future ZIKV outbreaks in Africa and to investigate possible ZIKV infections that have occurred in the past during the pregnancies of mothers of babies with microcephaly. 
